# Type 2 diabetes and cognitive impairment in an older population with overweight or obesity and metabolic syndrome: baseline cross-sectional analysis of the PREDIMED-plus study

**DOI:** 10.1038/s41598-018-33843-8

**Published:** 2018-10-31

**Authors:** Núria Mallorquí-Bagué, María Lozano-Madrid, Estefanía Toledo, Dolores Corella, Jordi Salas-Salvadó, Aida Cuenca-Royo, Jesús Vioque, Dora Romaguera, J. Alfredo Martínez, Julia Wärnberg, José López-Miranda, Ramón Estruch, Aurora Bueno-Cavanillas, Ángel Alonso-Gómez, Josep A. Tur, Francisco J. Tinahones, Lluís Serra-Majem, Vicente Martín, José Lapetra, Clotilde Vázquez, Xavier Pintó, Josep Vidal, Lidia Daimiel, José J. Gaforio, Pilar Matía, Emilio Ros, Roser Granero, Pilar Buil-Cosiales, Rocío Barragán, Mònica Bulló, Olga Castañer, Manoli García-de-la-Hera, Aina M. Yáñez, Itziar Abete, Antonio García-Ríos, Miguel Ruiz-Canela, Andrés Díaz-López, Susana Jiménez-Murcia, Miguel A. Martínez-González, Rafael De la Torre, Fernando Fernández-Aranda

**Affiliations:** 1 Department of Psychiatry, University Hospital of Bellvitge-IDIBELL, Barcelona, Spain; 2 CIBER de Fisiopatología de la Obesidad y la Nutrición (CIBEROBN), Instituto de Salud Carlos III, Madrid, Spain; 3 University of Navarra, Department of Preventive Medicine and Public Health, Medical School, and Navarra Institute for Health Research (IdiSNA), Pamplona, Spain; 4 Department of Preventive Medicine, University of Valencia, Valencia, Spain; 5 Rovira i Virgili University, Department of Biochemistry and Biotechnology, Human Nutrition Unit, IISPV, Hospital Universitari Sant Joan de Reus, Reus, Spain; 6 Instituto Hospital del Mar de Investigaciones Médicas, Barcelona, Spain; 7 University of Miguel Hernández, ISABIAL-FISABIO, Alicante, Spain; 8 CIBER de Epidemiología y Salud Pública (CIBERESP), Instituto de Salud Carlos III, Madrid, Spain; 9 Instituto de Investigación Sanitaria Illes Balears (IdISBa), University Hospital of Son Espases, Palma de Mallorca, Spain; 10 Department of Nutrition, Food Sciences, and Physiology, Center for Nutrition Research, University of Navarra, Pamplona, IMDEAfood, Madrid, Spain; 11 Department of nursing, school of health sciences. University of Malaga-IBIMA, Malaga, Spain; 12 Lipids and Atherosclerosis Unit, Department of Internal Medicine, Maimonides Biomedical Research Institute of Cordoba (IMIBIC), Reina Sofia University Hospital, University of Cordoba, Cordoba, Spain; 13 Department of Internal Medicine, Institut d’Investigacions Biomèdiques August Pi Sunyer (IDIBAPS), Hospital Clínic, University of Barcelona, Barcelona, Spain; 14 Departament of Preventive Medicine and Public Health, University of Granada, Granada, Spain; 15 Department of Cardiology, University Hospital Araba, Vitoria, Spain; 16 Research Group on Community Nutrition and Oxidative Stress, University of the Balearic Islands, Palma de Mallorca, Spain; 17 Department of Endocrinology and Nutrition, Virgen de la Victoria Hospital (IBIMA), Malaga University, Malaga, Spain; 18 Research Institute of Biomedical and Health Sciences, University of Las Palmas de Gran Canaria, Las Palmas de Gran Canaria, Spain; 19 Biomedicine Institute (IBIOMED), University of León, León, Spain; 20 Department of Family Medicine, Research Unit, Distrito Sanitario Atención Primaria Sevilla, Sevilla, Spain; 21 Department of Endocrinology and Nutrition, University Hospital Fundación Jiménez Díaz, Madrid, Spain; 22 Lipid Unit, Department of Internal Medicine, Bellvitge Biomedical Research Institute (IDIBELL)-Hospital Universitari de Bellvitge, L’Hospitalet de Llobregat, Barcelona, Spain; 23 Department of Endocrinology and Nutrition, Hospital Clínic, Barcelona, Spain; 24 CIBER de Diabetes y Enfermedades Metabólicas asociadas (CIBERDEM), Barcelona, Spain; 25 Nutritional Genomics and Epigenomics Group, IMDEA Food, CEI UAM + CSIC, 28049 Madrid, Spain; 26 Center for Advanced Studies in Olive Grove and Olive Oils, University of Jaén, Jaén, Spain; 27 Endocrinology and Nutrition Department, Hospital Clínico San Carlos-IdISSC, Facultad de Medicina, Universidad Complutense de Madrid, Madrid, Spain; 28 Lipid Clinic, Endocrinology and Nutrition Service, IDIBAPS, Hospital Clínic, University of Barcelona, Barcelona, Spain; 29 Departament de Psicobiologia i Metodologia, Universitat Autònoma de Barcelona, Barcelona, Spain; 30 Primary Health Care Division, Servicio Navarro de Salud-Osasunbidea, Pamplona, Spain; 31 Universitat de les Illes Balear, Palma de Mallorca, Spain; 32 Department of Clinical Sciences, School of Medicine, University of Barcelona, Barcelona, Spain; 33 Harvard TH Chan School of Public Health, Dpt. Nutrition, Harvard, University, Boston, USA; 34 Department of Experimental and Health Sciences, University Pompeu Fabra, Barcelona, Spain

## Abstract

This study cross-sectionally examines in the elderly population: (a) the association of type 2 diabetes with executive function (EF); (b) the effect of BMI on both type 2 diabetes and EF; (c) the association between glycaemia control and EF in type 2 diabetes. 6823 older individuals with overweight/obesity and metabolic syndrome participating in the PREDIMED-PLUS study, were assessed with a battery of cognitive tests and a medical interview. ANOVA showed a significantly worse performance on EF in type 2 diabetes vs. non-diabetic individuals. Two complementary models were displayed: (1) in the whole sample, the presence of type 2 diabetes, depressive symptoms and BMI had a direct negative effect on EF, while apnoea had an indirect negative effect; (2) in the diabetes subsample, higher illness duration was associated with worse performance in EF. Participants with type 2 diabetes and HbA1c<53 mmol/mol displayed better cognitive performance when compared to those with HbA1c≥53 mmol/mol. Our results provide a controlled comprehensive model that integrates relevant neuropsychological and physical variables in type 2 diabetes. The model suggests that, to improve treatment adherence and quality of life once diabetes has been diagnosed, cognitive decline prevention strategies need to be implemented while monitoring depressive symptoms, BMI and glycaemia control.

## Introduction

Type 2 diabetes is caused by a combination of resistance to insulin action and an inadequate compensatory insulin secretory response^1^. This is one of the most frequent diseases among the elderly individuals, with studies reporting a prevalence ranging from 19% to 33% in general population^2,3^. With global population ageing at significant pace and given the high prevalence of type 2 diabetes among the elderly, the negative effects of this chronic metabolic disease on health and cognitive functioning are of notable clinical interest.

With this regards, the main cognitive domains that have been related to type 2 diabetes are the following: attention, memory, processing speed and executive function (EF; i.e. working memory, cognitive flexibility, inhibitory control, etc.), as well as global cognitive functioning. When examining cognitive impairment in older individuals (>60 years), results confirmed that patients with type 2 diabetes in comparison with those without type 2 diabetes displayed higher impairment in global cognitive function, working memory and cognitive flexibility^4,5^. Similarly, when exploring other cohorts of elderly population (>65) with normal cognition, it was confirmed that type 2 diabetes participants obtained lower scores than non-diabetic patients in some cognitive domains such as attention, psychomotor function, information-processing speed and executive function, but they did not differ in memory^6,7^.

Different prospective studies have analyzed the association between the presence of type 2 diabetes and the risk of cognitive dysfunction. In older adults (42–89 years), results showed that women with type 2 diabetes had an increased risk of major cognitive decline in verbal fluency after 4 years when compared with non-diabetic women^8^. Similarly, other longitudinal studies concluded that older women (>65) with type 2 diabetes experienced an accelerated cognitive decline in general functioning and other specific domains, including visual and working memory as well as verbal fluency^5,9,10^. In addition, research in old population exploring the duration of type 2 diabetes point towards an association between the duration of this metabolic disease and a poorer performance in verbal memory and concept formation^11^ as well as in general cognition^10^. A longer duration of type 2 diabetes together with an earlier onset, has also been reported to increase the risk of mild cognitive impairment development^12^.

The increasing number of individuals suffering from type 2 diabetes among older people during the last few decades might be due to inappropriate dietary habits and a sedentary lifestyle, which lead to overweight and obesity. An elevated BMI may cause insulin resistance and it is common in adults with type 2 diabetes^13^. Some findings show that both high BMI and obesity are associated with a worsened performance in measures associated with EF, working memory, short-term memory and verbal fluency^14–16^.

Finally, type 2 diabetes has been associated with cognitive dysfunction regardless of ethnicity and cultural factors^17^. Nevertheless, other aspects seem to be relevant when examining the negative effects of type 2 diabetes on cognitive performance. In this line, different studies report that gender^8^, low glycaemia control^18,19^ as well as the presence of depressive symptoms^20^ are associated with worse cognitive performance in type 2 diabetes individuals. It has also to be note that sleep disorders can have an impact on cognition, especially in older age population at higher risk of cognitive decline^21^.

In light of previous studies and in order to specify the association between the presence of type 2 diabetes and cognitive decline in older individuals (above 55 years of age) with overweight/obesity and metabolic syndrome, the objectives of this study are: (a) to explore the associations of type 2 diabetes with cognitive performance (i.e.: EF); (b) to evaluate the effect of BMI on type 2 diabetes and cognitive performance when controlling for other confounding variables (e.g.: depressive symptoms, sleep disorders, education, age and sex); (c) to explore the association between glycaemia control and cognitive function performance in individuals with type 2 diabetes.

## Methods

This is a cross-sectional analysis on baseline data within the frame of the PREDIMED-Plus study, a 6-year multicenter, randomized, parallel-group, primary prevention clinical trial conducted in Spain to assess the effect of an intensive weight loss intervention program based on an energy-restricted traditional Mediterranean diet, physical activity promotion and behavioural support (intervention group), compared to a usual care intervention and energy-unrestricted Mediterranean diet recommendations (control group). A more detailed description of the PREDIMED-plus study and the study protocol is available at http://predimedplus.com/wp-content/uploads/2016/07/Protocolo_PREDIMED_PLUS_eng_23112016_adl.pdf. This study was registered at the International Standard Randomized Controlled Trial (ISRCT; http://www.isrctn.com/ISRCTN89898870) with number 89898870. All participants were recruited and randomized from October 2013 to December 2017 across 23 centres from different universities, hospitals and research institutes of Spain. Each of these centres recruited participants from several Primary Care Health Facilities belonging to the National Health System. The eligible participants were community-dwelling adults (aged between 55 and 75 years in case of men; and between 60 and 75 years in women) with overweight/obesity (BMI between 27 and 40 kg/m2), who met at least three components of the metabolic syndrome according to the updated harmonized criteria of the International Diabetes Federation and the American Heart Association and National Heart, Lung and Blood Institute^22^. Specific PREDIMED-plus exclusion criteria can be found in the website http://predimedplus.com/en. Participants included in the current analysis provided data on cognitive performance and depressive symptoms, as well as detailed specification of BMI, physical conditions and sociodemographic data. Additionally, glucose and HbA1c concentrations were measured at the same time point of the mood and cognitive assessment. All participants provided written informed consent, and the study protocol and procedures were approved according to the ethical standards of the Declaration of Helsinki by the Research Ethics Committees from all the participating institutions (Supplementary Info 1).

Exclusion criteria for the present analysis were: (1) reported dementia (2) head trauma, learning disability or intellectual disabilities; (3) presence of depression or a lifetime mental disorder according to DSM-5. Finally, all the included participants presented glucose levels within the normal range during the neuropsychological assessment.

### Instruments/measures

All participants completed a self-report questionnaire for exploring depressive symptoms as well as a set of neuropsychological tests for assessing different cognitive domains that included attention, short term memory, verbal fluency, cognitive flexibility, visuospatial skills and working memory. Sociodemographic information was also collected by means of a self-reported questionnaire and a hetero-administered clinical interview assessing medical conditions (including current presence of type 2 diabetes and years of duration since its onset, presence of apnoea, etc.).

#### Clinical and neuropsychological assessment

-The Beck Depression Inventory–II (BDI-II)^23^ is a 21-item self-report measure for assessing the severity of depressive symptoms in adults and adolescents (ages from 13 to 80 years). The BDI-II reflects the diagnostic criteria for Major Depressive Disorder listed in the Diagnostic and Statistical Manual of Mental Disorders (4th ed.)^24^. Scores for each item range from 0 to 3; the total score is the sum of all responses. The Cronbach’s alpha in our sample shows a good internal consistency (α = 0.883).

The Trail Making Test (TMT)^25^ consists of 25 circles spread out over two sheets of paper (parts A and B). In the part A, participants are requested to connect consecutive numbers (1–2–3–4-…) in the correct order by drawing a line. In the part B, they have to connect consecutive numbers and letters in an alternating numeric and alphabetic sequence (1-A, 2-B, 3-C-…). Each part is scored according to the time spent to complete the task (lower score mean better performance). TMT is frequently used to assess executive function. More specifically, TMT-A is used for measuring attention, psychomotor speed and visuospatial skills; while, TMT-B is used for measuring divided attention, cognitive flexibility and interference.

The Verbal Fluency Test^26^ assess verbal ability and executive control. Participants need to retrieve words of their language by accessing their mental lexicon, but also focus on the task and avoid repetition that involves executive control processes. In the phonemic fluency task, participants are requested to produce as many words as possible that start with the letter P during 60 seconds. Participants were asked to avoid producing names of people or places and repetitions of the same word with a different suffix. Secondly, in the semantic fluency task, participants have to name as many different animals as they can without repeating them during 60 seconds. The total raw score for each of the tasks corresponds to the number of words the participant achieves to say.

The Digit Span (DS) test of the Wechsler Adult Intelligence Scale-III (Spanish version)^27^ is compound of two different subtests: DS Forward, considered as a test of attention and short-term memory capacity; and DS Backward recall, considered as a test of working memory capacity. The DS Forward recall requires participants to repeat orally a series of random single digits in the same order they heard them; the sequence of digits varies in length from three to nine. Contrary, in the DS Backward recall participants are asked to repeat a series of random single digits in reverse order; the sequence varies from two to eight. The performance on the DS can be reported by a direct score of the backward performance (ranging from 1 to 16) and the forward performance (ranging from 1 to 14) and, by the span of immediate recall (backward span range from 1 to 8 and forward span range from 1 to 7).

#### Anthropometric and biochemical measurements

Weight and height were measured with light clothing and no shoes with calibrated scales and a wall-mounted stadiometer, respectively. BMI was calculated as weight in kilograms divided by the square of height in meters. Serum glucose levels were measured using standard enzymatic methods. HbA1c was measured by standard routine methods and glycaemia control was specified (HbA1c<53 mmol/mol [7%])^1^.

### Statistical analysis

Analyses were carried out with Stata15 for Windows. We used the study baseline database generated in August 2017. The comparisons of the cognitive measures between participants with and without type 2 diabetes was done with ANOVA procedures adjusted for the individuals’ sex, age, years of school attendance and presence of sleep apnoea syndrome. Cohen’s-*d* coefficients measured the effect size for the mean comparisons (low effect size was considered low for |*d*|> 0.2, moderate for |*d*|> 0.5 and large for |*d*|> 0.8)^28^. The Finner’s method, a procedure included into the Familywise error rate stepwise procedures which offers a more powerful test than the classical Bonferroni correction^29^, was used to control Type-I error due to multiple comparisons. The same method was used to explore the association between glycaemia control (HbA1c<53 mmol/mol [7%]) and cognitive function performance in the subsample of participants who presented type 2 diabetes.

The underlying mechanisms between BMI, type 2 diabetes and cognitive measures were examined with path-analysis modelling, a straightforward extension of multiple regression techniques that can be used for both confirmatory and exploratory modelling. The aim of this procedure is to estimate the magnitude and significance of hypothesized connections/relationships into a set of variables, and so they allow to theory testing and theory development^30^. The Path analysis used in this study was implemented through Structural Equation Modelling (SEM) with exploratory aims, with the advantage (compared to classical regression models) to allow the inclusion of multiple relationships among a set of variables, including mediational associations. The maximum-likelihood estimation method of parameter estimation was used and goodness-of-fit was evaluated using standard statistical measures^31^: the root mean square error of approximation (RMSEA), Bentler’s Comparative Fit Index (CFI), the Tucker-Lewis Index (TLI), and the standardized root mean square residual (SRMR). Adequate model fit was considered if the following criteria were met: RMSEA< 0.08, TLI> 0.9, CFI> 0.9 and SRMR< 0.1; the global predictive capacity of the model was measured by the coefficient of determination. Separate models were obtained for the whole sample (*n* = 6,825) and for the type 2 diabetes sub-sample (*n* = 1,860).

## Results

The initial sample was constituted of 6865 participants. According to the exclusion criteria of this study, for the present analyses forty nine participants were excluded for presenting hypo or hyperglycaemia during the cognitive assessment (≤60 mmol/l, hypoglycaemia; ≥150 mmol/l, hyperglycaemia). The final sample for the present analysis consisted of 6823 participants (mean age: 64.95 years; 48.6% females) distributed as follow: 4964 with no type 2 diabetes and 1859 with reported type 2 diabetes. At the moment of the assessment most of the patients with type 2 diabetes had presented this affection for more than 5 years (55.4%), some of them during 1 to 5 years (32.2%) and a 10.4% of them were only diagnosed during the last year (see the Supplementary Figure S1 for the flowchart graphic of the sample which is available as online supporting material).

Table 1 includes the descriptive data for the categorical variables (top of the table) and quantitative measures (bottom part of the table), for the whole sample and stratified by the sub-samples defined by the presence-absence of type 2 diabetes. No differences between groups were found for the origin or the civil status. However, the subsample who presented type 2 diabetes included a higher proportion of men, non-employed, with lower level studies, older age, a higher prevalence of sleep apnoea and depression symptomatology, higher BMI and higher concentrations of HbA1c and glucose.

**Table 1:** Descriptive variables of the studied simple.

	Total (*n* = 6,823)	Type 2 diabetes = absent (*n* = 4,964)	Type 2 diabetes = present (*n* = 1,859)	χ^2^	*df*	*p*
	*n (*%)	*n (*%)	*n (*%)			
Sex						
*Male*	3506 (51.4%)	2485 (50.1%)	1021 (54.9%)	12.78	1	0.001
*Female*	3317 (48.6%)	2479 (49.9%)	838 (45.1%)			
Origin						
*Europe*	6651 (97.5%)	4825 (97.2%)	1826 (98.2%)	6.95	5	0.256
*South-America*	153 (2.2%)	124 (2.5%)	29 (1.6%)			
*North-Africa*	10 (0.1%)	7 (0.1%)	3 (0.2%)			
*South-Sahara*	6 (0.1%)	5 (0.1%)	1 (0.1%)			
*Asian*	2 (0.0%)	2 (0.0%)	0 (0.0%)			
*Other*	1 (0.0%)	1 (0.0%)	0 (0.0%)			
Civil status						
*Single*	340 (5.0%)	257 (5.2%)	83 (4.5%)			
*Married*	5230 (76.7%)	3778 (76.1%)	1452 (78.1%)	5.99	5	0.308
*Widow*	711 (10.4%)	529 (10.7%)	182 (9.8%)			
*Divorced*	386 (5.7%)	278 (5.6%)	108 (5.8%)			
*Separated*	154 (2.3%)	120 (2.4%)	34 (1.8%)			
*Religious*	2 (0.0%)	2 (0.0%)	0 (0.0%)			
Education						
*University* (*high)*	896 (13.1%)	681 (13.7%)	215 (11.6%)	17.22	4	0.004
*University* (*grade)*	613 (9.0%)	465 (9.4%)	148 (8.0%)			
*Secondary*	1968 (28.8%)	1449 (29.2%)	519 (27.9%)			
*Primary or less*	3346 (49.1%)	2369 (47.7%)	977 (42.6%)			
Employment						
*Employed*	1412 (20.7%)	1085 (21.9%)	327 (17.6%)	19.27	7	0.013
*Total incapacity*	110 (1.6%)	71 (1.4%)	39 (2.1%)			
*Work at home*	1009 (14.8%)	736 (14.8%)	273 (14.7%)			
*Student*	51 (0.7%)	36 (0.7%)	15 (0.8%)			
*Retired*	3800 (55.7%)	2723 (54.9%)	1077 (57.9%)			
*Work leave* (*>3 months)*	55 (0.8%)	37 (0.7%)	18 (1.0%)			
*Unemployed* (*incomes)*	253 (3.7%)	180 (3.6%)	73 (3.9%)			
*Unemployed* (*no-incomes)*	133 (1.9%)	96 (1.9%)	37 (2.0%)			
Weight group						
*Over-weight:BMI ⊂* (*25–30]*	1831 (26.8%)	1355 (27.3%)	476 (25.6%)	5.31	4	0.204
*Obesity I: BMI ⊂* (*30–35]*	3351 (49.1%)	2449 (49.3%)	902 (48.5%)			
*Obesity II: BMI ⊂* (*35–40]*	1589 (23.3%)	1122 (22.6%)	467 (25.1%)			
*Obesity III: BMI>40*	52 (0.8%)	38 (0.8%)	14 (0.8%)			
Presence of sleep apnoea						
*No*	5948 (87.2%)	4374 (88.1%)	1574 (84.7%)	14.36	1	0.001
*Yes*	875 (12.8%)	590 (11.9%)	285 (15.3%)			
Tobacco use (at current)						
*No*	5975 (87.6%)	4351 (87.7%)	1624 (87.4%)	0.11	1	0.745
*Yes*	848 (12.4%)	613 (12.3%)	235 (12.6%)			
Alcohol use						
*No*	1432 (21.0%)	1014 (20.4%)	418 (22.5%)	3.46	1	0.126
*Yes*	5391 (79.0%)	3950 (79.6%)	1441 (77.5%)			
	*Mean* ± *SD*	*Mean* ± *SD*	*Mean* ± *SD*	*F*	*df*	*p*
Age (years-old)	64.95 ± 4.91	64.88 ±4.93	65.15 ± 4.86	4.23	1;6,821	0.040
Education level (years)	11.37 ± 5.23	11.53 ± 5.24	10.96 5.19	16.34	1;6,821	<0.001
BMI (kg/m^2^)	32.56 ± 3.44	32.49 ± 3.42	32.73 3.50	6.04	1;6,821	0.017
Glycated hemoglobin A1c, in %	6.14 ± 2.25	5.87 ± 2.51	6.87 1.01	239.69	1;6,821	<0.001
Plasma glucose levels (mg/dl)	112.48 ± 26.10	103.50 ± 15.21	136.54 33.03	3112.5	1;6,821	<0.001
Depression (BDI raw total)	8.47 ± 7.42	8.20 ± 7.22	9.20 ± 7.88	24.58	1;6,821	<0.001

*Note*. *p-value* includes Finner’s correction for multiple comparisons. SD, standard deviation.

### Comparison of cognitive measures between participants with and without type 2 diabetes

Table 2 includes the results obtained in the ANOVA procedures adjusted for sex, age, years of school attendance and presence of apnoea. Some significant results emerged comparing patients with and without type 2 diabetes: the presence of type 2 diabetes was associated with lower scores in the phonological and semantic verbal fluency, and to higher scores in the trail making test.

**Table 2:** Comparison of the cognitive assessment variables between patients with and without reported type 2 diabetes.

	Type 2 diabetes = absent (*n* = 4,964)		Type 2 diabetes = present (*n* = 1,859)		*MD*	*F*_*1;6817*_	*p*	*95% CI* (*MD*)		*|d|*
	*Mean*	*SD*	*Mean*	*SD*						
Phonological verbal fluency of letter P: total	12.40	4.59	11.74	4.39	0.66	33.93	**<0**.001*	0.44	0.88	0.15
Semantic verbal fluency of animals: total	16.22	4.96	15.63	4.74	0.58	22.63	**<0**.001*	0.34	0.82	0.12
Trail Making Test: A, total time (seconds)	52.14	27.66	54.02	28.74	−1.88	7.00	**0**.016*	−3.26	−0.49	0.07
Trail Making Test: B, total time (seconds)	123.78	63.84	130.70	69.63	−6.92	17.91	**<0**.001*	−10.13	−3.72	0.10
Digit: forward score	8.81	2.46	8.80	2.49	0.02	0.07	0.798	−0.13	0.16	0.01
Digit: backwards core	5.16	2.27	5.20	2.14	−0.04	0.29	0.637	−0.16	0.09	0.02
Digit: forward Span	5.70	1.35	5.61	1.33	0.10	2.94	0.114	−0.01	0.21	0.07
Digit: backwards Span	3.81	1.25	3.72	1.19	0.09	3.24	0.115	−0.01	0.19	0.07

*Note*. SD, standard deviation; MD, mean difference; |*d*|, Cohen’s-*d* measuring effect size.

*Bold: significant comparison (0.05 level, including Finner’s correction).

All the results adjusted for sex, age, education and presence of apnoea.

Table 3 includes the ANOVA procedures exploring the relationship between glycaemia control (HbA1c<53 mmol/mol [7%] versus HbA1c≥53 mmol/mol [7%]) and cognitive function performance in the subsample of participants who presented type 2 diabetes. Statistical differences emerged for the measures of phonological fluency and digit backward, achieving better functioning (higher means) the participants into the HbA1c<53 mmol/mol (7%) group.

**Table 3:** Comparison for type 2 diabetes patients with HbA1c higher and lower than 53 mmol/mol (7%).

Type 2 diabetes = present (*n* = 1,620)	HbA1c<53 mmol/mol (7%) (*n* = 979)		HbA1c≥53 mmol/mol (7%) (*n* = 641)		*MD*	*F*_*1;1615*_	*p*	*95% CI* (*MD*)		*|d|*
	*Mean*	*SD*	*Mean*	*SD*						
Phonological verbal fluency of letter P: total	11.90	4.44	11.35	4.35	0.55	7.16	**0**.036*	0.15	0.95	0.12
Semantic verbal fluency of animals: total	15.68	4.71	15.38	4.84	0.30	1.82	0.269	−0.14	0.74	0.06
Trail Making Test: A, total time (seconds)	54.32	28.45	53.92	27.86	0.40	0.09	0.807	−2.20	3.00	0.01
Trail Making Test: B, total time (seconds)	129.88	69.78	132.51	68.98	−2.63	0.68	0.507	−8.90	3.64	0.04
Digit: forward score	8.76	2.48	8.54	2.41	0.22	2.50	0.244	−0.05	0.49	0.09
Digit: backwards core	5.20	2.25	4.87	1.93	0.33	8.13	**0**.035*	0.10	0.56	0.16
Digit: forward Span	5.68	1.39	5.52	1.29	0.16	2.72	0.244	−0.03	0.34	0.12
Digit: backwards Span	3.71	1.25	3.70	1.15	0.01	0.02	0.888	−0.15	0.18	0.01
HbA1c	6.23	0.44	7.84	0.83	−1.61	257.2	**<0**.001*	−1.67	−1.55	2.42

*Note*. SD, standard deviation; MD, mean difference; |*d*|, Cohen’s-*d* measuring effect size.

*Bold: significant comparison (0.05 level, including Finner’s correction).

All the results adjusted for sex, age, education and presence of apnea.

### SEM examining the association between BMI, type 2 diabetes and cognitive functioning

**Figure 1:**
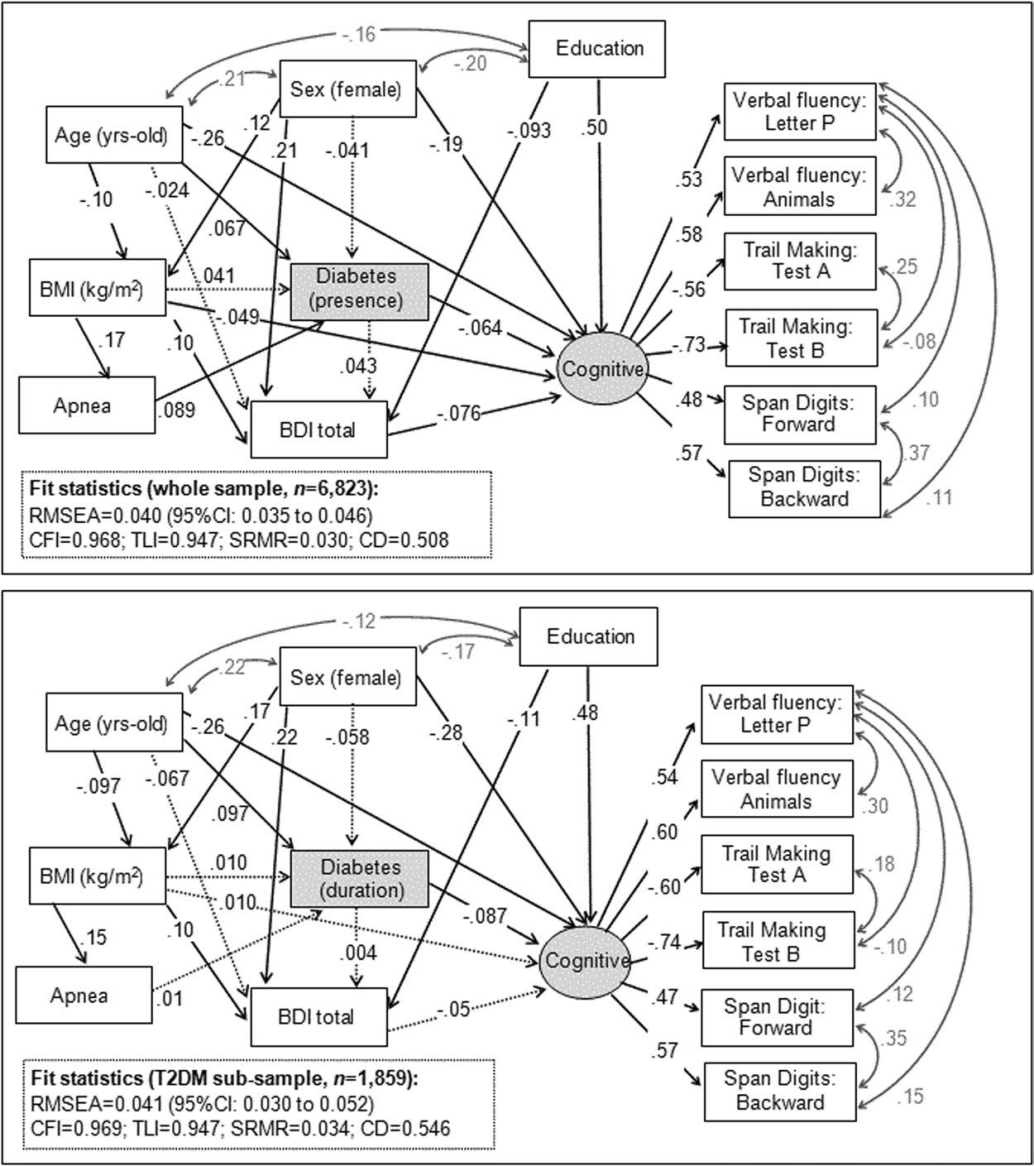
SEM model for the presence of type 2 diabetes (first panel) and for the duration of type 2 diabetes (second panel). Note. Continuous line refers to significant coefficients (<0.05 level). Dash-line refers to non-significant coefficients. Grey font: covariance parameter.

Figure 1 shows the standardized coefficients of the SEM obtained for the whole sample (first panel, *n* = 6,823) and for the subsample with type 2 diabetes (*n* = 1,859). Both models included a latent variable (labelled *cognitive*) which was defined by the cognitive scores analyzed in this study (as high the score in this variable the better the neuropsychological functioning). Goodness-of-fit was obtained for both models, as well as very good global predictive capacity. Supplementary Info S2 and S3 contain the complete results of the path-analyses, including the parameters estimating the direct, indirect and total effects. contain the complete results of the path-analyses, including the parameters estimating the direct, indirect and total effects.

In the whole sample, the presence of type 2 diabetes was predictive of worse cognitive functioning (direct negative effect); while the BMI directly contributed on the cognitive performance (as high the BMI as poor the cognitive performance). In the subsample with type 2 diabetes, the higher duration of this metabolic condition was predictive of a lower cognitive functioning (direct effect), while BMI did not contributed to (with direct or indirect effects) on the cognitive levels.

Regarding the remaining variables included in the SEM, for both models better cognitive functioning was directly related to male sex, younger age and higher education. The apnoea achieved indirect effects on the cognitive performance only in the whole sample, through one path-way: the presence of apnoea was directly related to type 2 diabetes, which was also a predictor of poor cognitive execution. No direct effect between the presence of apnoea and cognitive performance emerged in any of the SEM.

As a synthesis of the results of the study, Figure S2 (Supplementary Material) includes the scheme of the main direct and indirect effects on the cognitive construct analyzed in the study.

## Discussion

The present study examined the effect of type 2 diabetes on cognitive performance specifically with EF tasks, in a large cohort of 6823 patients above 55 years of age. Additionally, the role that BMI, apnoea, age, sex, depressive symptoms and education play in these effects was also explored or controlled when appropriate. Mainly, results display worse cognitive functioning when participants present type 2 diabetes, with a larger effect on those who have a longer duration of this metabolic condition. Additionally, some other variables are also found to directly or indirectly worsen cognitive function among this population, namely BMI, sex, depressive symptoms and education.

Within the first analysis when comparing patients presenting type 2 diabetes with individuals without type 2 diabetes and controlling for other co-related variables that could interfere with the cognitive assessment (i.e.: sex, age, education and apnoea), results show a significantly worse cognitive performance on EF when type 2 diabetes is present. Thus, the presence of type 2 diabetes in patients above 55 years of age seems to have a clear impact on cognitive function which would not be explained by the education, age, sex or the presence of apnoea. In the same line, previous studies have also reported that type 2 diabetes has a negative impact on cognitive function and physical health^4–10,32,33^; taken together, our results add some extra evidence to previous findings and point towards the need to target these neurocognitive domains once type 2 diabetes has been diagnosed and its treatment protocol implemented. In an attempt to further explore the underlying mechanisms of the reported association between type 2 diabetes and cognitive performance, the present study sought to step further, by exploring the association of glycated haemoglobin with the neuropsychological measures in the subsample with type 2 diabetes. Previous studies have found that individuals with a weighted mean HbA1c less than 53 mmol/mol (7%) performed significantly better on tests of motor speed and psychomotor efficiency than those subjects whose time weighted mean HbAlc was greater^34^. In our study, individuals with type 2 diabetes and HbA1c<53 mmol/mol (7%) displayed better cognitive function when compared to those with HbA1c≥53 mmol/mol (7%), specifically on verbal fluency and working memory which highlights the importance of maintain good glycaemia control in this population which is already at risk of cognitive decline.

Additionally, we sought to assess the associations of the presence of type 2 diabetes and its duration, cognitive performance and BMI in a model that also took into account other variables reported to be associated with cognitive performance (i.e.: sex, mood, education and apnoea). As a result of this analysis, two complementary different models were obtained. These two models provide relevant information about the interaction and contribution of the described variables to the cognitive performance of population suffering from type 2 diabetes, which can help improving the current understanding and treatment of this affection. Firstly, it is important to note that, as expected, type 2 diabetes and a higher duration of this metabolic condition was associated with worse cognitive performance. Consequently, it seems highly important to implement cognitive stimulation protocols within the treatment of type 2 diabetes that could help to reduce the progression of the cognitive decline once type 2 diabetes has been diagnosed. Secondly, a high BMI has previously been linked to specific cognitive impairments in different sample populations, especially in extreme weight conditions^14–16,35^. Our findings with the whole sample model give extra evidence on the negative impact of higher BMI on cognitive performance with this older age sample population. Additionally, higher BMI also negatively affects the participants’ mood in our sample. In turn, the lower mood in our sample is linked to poorer cognitive performance, which has also been reported in another study conducted with clinically depressed individuals^36^. Thus, health programs should give special emphasis in promoting weight loss within these individuals as well as strategies to monitor and improve mood when needed. On the contrary, in our older age sample with overweight/obesity, BMI differences are not significant enough to be robustly linked to the presence or duration of type 2 diabetes. However, BMI is described as a risk factor for the occurrence of type 2 diabetes in older age population^37^. Thus, the results observed regarding the influence of BMI on type 2 diabetes may most certainly only be circumscribed to our sample.

With regards to the presence of sleep disturbances/disorders and cognitive decline^21^, in our sample sleep apnoea (which unsurprisingly presents an association with higher BMI) is only linked to cognitive decline through its role in type 2 diabetes. Finally, it is also relevant that as reported in older adult populations a better cognitive function is directly related to males, younger age and higher education^38,39^.

The present study should be considered under some limitations. Firstly, the cross-sectional design does not imply causality. Also, given that our study participants are senior adults with a metabolic syndrome the present findings cannot be extrapolated to other population groups. However, it is one of the strengths of this study that it is conducted with a large sample of men and women and analyses are adjusted for potential confounding factors. Thus, although the results cannot be generalizable to other populations, they are representative of individuals over 55 years of age who suffer from a metabolic syndrome. Future studies should further explore the associations reported regarding the presence of type 2 diabetes, cognitive decline and BMI in different sample populations. Cognitive dysfunction is of particular importance because of its impact on quality of life. Future studies should test if an early intervention for improving cognitive deficits as well as depressive symptoms would help to reduce de cognitive decline and to improve the type 2 diabetes treatment adherences as well as self-care and quality of life.

To conclude, in this cross-sectional assessment type 2 diabetes (including illness duration), high BMI and lower mood was linked to poorer cognitive function in older individuals with overweight/obesity and metabolic syndrome. A good glycaemia control in type 2 diabetes was associated with a better cognitive performance. Our results provide a controlled comprehensive model that integrates different neuropsychological and physical variables that are relevant in type 2 diabetes. The model also suggests that, to improve treatment adherence and quality of life once type 2 diabetes has been diagnosed, cognitive decline prevention strategies need to be implemented while closely monitoring depressive symptoms, BMI and glycaemia control. However, the etiopathogenic factors associated with the reported associations are still unclear.

## Electronic supplementary material

Supplementary Information
