# Supplementary material for: Type 2 diabetes and cognitive impairment in an older population with overweight or obesity and metabolic syndrome: baseline cross-sectional analysis of the PREDIMED-plus study
Source: Sci Rep. 2018 Oct 31;8:16128. doi: 10.1038/s41598-018-33843-8 (PMC6208341; doi:10.1038/s41598-018-33843-8)
Supplement: Supplementary file 1 — Supplementary Information [file 41598_2018_33843_MOESM1_ESM.docx]

**Type 2 diabetes and cognitive impairment in an older population with overweight or obesity and metabolic syndrome: baseline cross-sectional analysis of the PREDIMED-plus study**

**Supplementary Info S1.** Ethics committees of all the participating institutions.

CEI Provincial de Màlaga, CEI de los Hospitales Universitarios Virgen Macarena y Virgen del Rocío, CEI de la Universidad de Navarra/CEIC de Navarra, CEI de las Illes Balears, CEIC del Hospital Clínic de Barcelona, CEIC del Parc de Salut Mar, CEIC del Hospital Universitari Sant Joan de Reus, CEI del Hospital Universitario San Cecilio, CEIC de la Fundación Jiménez Díaz, CEIC Euskadi, CEI IMDEA Alimentación, CEI en Humanos de la Universidad de Valencia, CEIC del Hospital Universitario de Gran Canaria Doctor Negrín, CEIC del Hospital Universitario de Bellvitge, CEI de Córdoba, CEI de Instituto Madrileño De Estudios Avanzados, CEIC del Hospital Clínico San Carlos, CEI Provincial de Málaga, CEI de las Illes Balears, CCEI de la Investigación Biomédica de Andalucía and CEIC de León.

**Supplementary Figure S1**. Sample flowchart


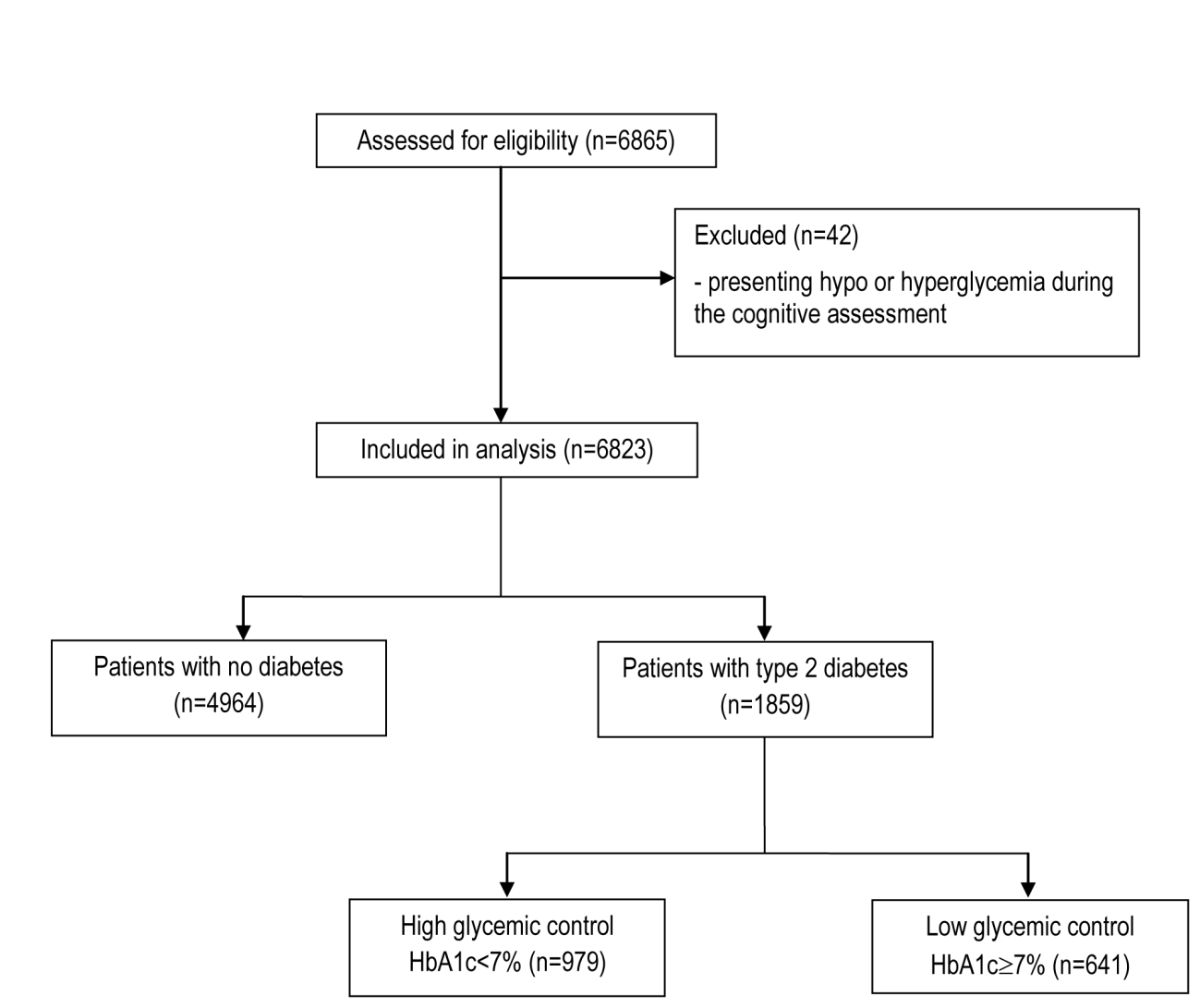


**Supplementary Figure S2.** Synthesis of the results measuring the contribution of the diabetes measures on the cognitive profile and the other variables of the study

**
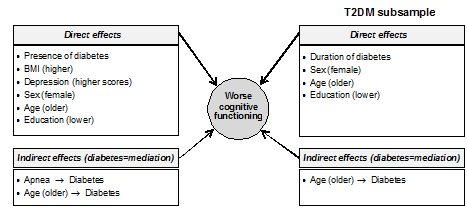
**

**Supplementary Info S2**. Complete results for the SEM obtained in the complete sample

**Direct effects Coef. Std. Err. z P> z Std. Coef.**

**--------------------------------------------------------------------------------------**

***Structural***

BDI-total

DIABETES .6646041 .3583968 1.85 0.064 .0402466

BMI .2289717 .0507286 4.51 0.000 .0988813

Sex 3.313922 .3606792 9.19 0.000 .2080793

Education-level -.1440416 .0345494 -4.17 0.000 -.0929236

Age -.0388312 .0364284 -1.07 0.286 -.0239382

------------------------------------------------------------------------------------

BMI

Sex .855159 .1563816 5.47 0.000 .1243372

Age -.0709379 .015928 -4.45 0.000 -.1012645

------------------------------------------------------------------------------------

APNEA-present

BMI .0163776 .0021004 7.80 0.000 .1723895

------------------------------------------------------------------------------------

Cognitive

DIABETES -.2963845 .1015796 -2.92 0.004 -.0640547

BDI-total -.0211942 .0063544 -3.34 0.001 -.0756392

BMI -.0320161 .0143661 -2.23 0.026 -.0493436

Sex -.8630164 .1071894 -8.05 0.000 -.1933905

Education-level .2179337 .0140633 15.50 0.000 .5017551

Age -.116206 .01124 -10.34 0.000 -.2556636

--------------------------------------------------------------------------------------

DIABETES

BMI .0057814 .0032157 1.80 0.072 .0412288

APNEA-present .1314918 .0340079 3.87 0.000 .089085

Sex -.0495741 .0225503 -2.20 0.068 -.0414015

Age .0066217 .0022503 2.94 0.003 .0674087

--------------------------------------------------------------------------------------

***Measurement***

Letter-P

Cognitive 1 (constrained) .5257817

------------------------------------------------------------------------------------

Animals

Cognitive 1.224351 .0622074 19.68 0.000 .5799018

------------------------------------------------------------------------------------

Test_A

Cognitive -6.162635 .3976206 -15.50 0.000 -.5571531

------------------------------------------------------------------------------------

Test_B

Cognitive -20.04489 1.073385 -18.67 0.000 -.7290418

------------------------------------------------------------------------------------

Digits-Forward

Cognitive .292784 .0186298 15.72 0.000 .4839235

------------------------------------------------------------------------------------

Digits-Backward

Cognitive .3135962 .0181067 17.32 0.000 .5704934

**Indirect effects Coef. Std. Err. z P> z Std. Coef.**

--------------------------------------------------------------------------------------

***Structural***

BDI-total

BMI .0052736 .0035384 1.49 0.136 .0022774

APNEA-present .08739 .0522659 1.67 0.095 .0035854

Sex .1673698 .0612739 2.73 0.006 .0105091

Age -.012216 .0058884 -2.07 0.038 -.0075308

------------------------------------------------------------------------------------

APNEA-present

Sex .0140054 .0031282 4.48 0.000 .0214344

Age -.0011618 .0003004 -3.87 0.000 -.0174569

------------------------------------------------------------------------------------

Cognitive

DIABETES -.0140858 .008691 -1.62 0.105 -.0030442

BMI -.0073164 .0022259 -3.29 0.001 -.0112762

APNEA-present -.0408243 .0170589 -2.39 0.017 -.0059775

Sex -.0884803 .0276724 -3.20 0.001 -.0198273

Education-level .0030528 .0011722 2.60 0.009 .0070287

Age .0015573 .0017249 0.90 0.367 .0034262

--------------------------------------------------------------------------------------

DIABETES

BMI .0021535 .0006217 3.46 0.001 .0153573

Sex .0067856 .0029798 2.28 0.023 .0070358

Age -.0005629 .0002578 -2.18 0.029 -.0057302

--------------------------------------------------------------------------------------

***Measurement***

Letter-P

DIABETES -.3104703 .1018976 -3.05 0.002 -.0352794

BDI-total -.0211942 .0063544 -3.34 0.001 -.0397697

BMI -.0393326 .0143985 -2.73 0.006 -.0318727

APNEA-present -.0408243 .0170589 -2.39 0.017 -.0031429

Sex -.9514967 .1062156 -8.96 0.000 -.112106

Education-level .2209865 .0141603 15.61 0.000 .2675092

Age -.1146486 .0112057 -10.23 0.000 -.1326218

------------------------------------------------------------------------------------

Animals

DIABETES -.3801247 .1234993 -3.08 0.002 -.0389108

BDI-total -.0259492 .0077335 -3.36 0.001 -.0438633

BMI -.0481569 .0174706 -2.76 0.006 -.0351535

APNEA-present -.0499833 .0207563 -2.41 0.016 -.0034664

Sex -1.164966 .1306536 -8.92 0.000 -.1236453

Education-level .2705652 .0148603 18.21 0.000 .2950446

Age -.1403702 .0134986 -10.40 0.000 -.1462729

------------------------------------------------------------------------------------

Test_A

DIABETES 1.913315 .6239583 3.07 0.002 .0373843

BDI-total .1306123 .0390848 3.34 0.001 .0421426

BMI .2423923 .088553 2.74 0.006 .0337745

APNEA-present .2515853 .1047152 2.40 0.016 .0033304

Sex 5.863727 .6685324 8.77 0.000 .1187949

Education-level -1.361859 .0772691 -17.62 0.000 -.2834705

Age .7065377 .0693076 10.19 0.000 .1405348

------------------------------------------------------------------------------------

Test_B

DIABETES 6.223344 2.017443 3.08 0.002 .0489179

BDI-total .424836 .1260282 3.37 0.001 .0551442

BMI .7884172 .2869344 2.75 0.006 .0441943

APNEA-present .818319 .3393592 2.41 0.016 .0043578

Sex 19.07265 2.060045 9.26 0.000 .1554446

Education-level -4.429652 .2144735 -20.65 0.000 -.3709247

Age 2.29812 .209628 10.96 0.000 .1838916

------------------------------------------------------------------------------------

Digits-Forward

DIABETES -.0909007 .0296891 -3.06 0.002 -.0324707

BDI-total -.0062053 .0018518 -3.35 0.001 -.0366036

BMI -.0115159 .0042077 -2.74 0.006 -.0293353

APNEA-present -.0119527 .0049796 -2.40 0.016 -.0028927

Sex -.278583 .0325507 -8.56 0.000 -.1031811

Education-level .0647013 .0041287 15.67 0.000 .2462124

Age -.0335673 .0033032 -10.16 0.000 -.1220636

------------------------------------------------------------------------------------

Digits-Backward

DIABETES -.0973623 .0316829 -3.07 0.002 -.0382795

BDI-total -.0066464 .0019743 -3.37 0.001 -.0431517

BMI -.0123345 .004477 -2.76 0.006 -.0345832

APNEA-present -.0128023 .0053216 -2.41 0.016 -.0034101

Sex -.2983858 .0338224 -8.82 0.000 -.1216393

Education-level .0693005 .0039647 17.48 0.000 .2902578

Age -.0359534 .0034189 -10.52 0.000 -.1438998

**Total effects Coef. Std. Err. z P> z Std. Coef.**

--------------------------------------------------------------------------------------

***Structural***

BDI-total

DIABETES .6646041 .3583968 1.85 0.064 .0402466

BMI .2342453 .0506931 4.62 0.000 .1011587

APNEA-present .08739 .0522659 1.67 0.095 .0035854

Sex 3.481292 .3598995 9.67 0.000 .2185883

Education-level -.1440416 .0345494 -4.17 0.000 -.0929236

Age -.0510472 .0363767 -1.40 0.161 -.031469

------------------------------------------------------------------------------------

BMI

Sex .855159 .1563816 5.47 0.000 .1243372

Age -.0709379 .015928 -4.45 0.000 -.1012645

------------------------------------------------------------------------------------

APNEA-present

BMI .0163776 .0021004 7.80 0.000 .1723895

Sex .0140054 .0031282 4.48 0.000 .0214344

Age -.0011618 .0003004 -3.87 0.000 -.0174569

------------------------------------------------------------------------------------

Cognitive

DIABETES -.3104703 .1018976 -3.05 0.002 -.0670989

BDI-total -.0211942 .0063544 -3.34 0.001 -.0756392

BMI -.0393326 .0143985 -2.73 0.006 -.0606197

APNEA-present -.0408243 .0170589 -2.39 0.017 -.0059775

Sex -.9514967 .1062156 -8.96 0.000 -.2132177

Education-level .2209865 .0141603 15.61 0.000 .5087838

Age -.1146486 .0112057 -10.23 0.000 -.2522374

--------------------------------------------------------------------------------------

Structural

DIABETES

BMI .0079349 .003168 2.50 0.012 .0565861

APNEA-present .1314918 .0340079 3.87 0.000 .089085

Sex -.0427885 .0224137 -1.91 0.056 -.0443657

Age .0060588 .0022428 2.70 0.007 .0616785

--------------------------------------------------------------------------------------

***Measurement***

Letter-P

DIABETES -.3104703 .1018976 -3.05 0.002 -.0352794

BDI-total -.0211942 .0063544 -3.34 0.001 -.0397697

BMI -.0393326 .0143985 -2.73 0.006 -.0318727

APNEA-present -.0408243 .0170589 -2.39 0.017 -.0031429

Cognitive 1 (constrained) .5257817

Sex -.9514967 .1062156 -8.96 0.000 -.112106

Education-level .2209865 .0141603 15.61 0.000 .2675092

Age -.1146486 .0112057 -10.23 0.000 -.1326218

------------------------------------------------------------------------------------

Animals

DIABETES -.3801247 .1234993 -3.08 0.002 -.0389108

BDI-total -.0259492 .0077335 -3.36 0.001 -.0438633

BMI -.0481569 .0174706 -2.76 0.006 -.0351535

APNEA-present -.0499833 .0207563 -2.41 0.016 -.0034664

Cognitive 1.224351 .0622074 19.68 0.000 .5799018

Sex -1.164966 .1306536 -8.92 0.000 -.1236453

Education-level .2705652 .0148603 18.21 0.000 .2950446

Age -.1403702 .0134986 -10.40 0.000 -.1462729

------------------------------------------------------------------------------------

Test_A

DIABETES 1.913315 .6239583 3.07 0.002 .0373843

BDI-total .1306123 .0390848 3.34 0.001 .0421426

BMI .2423923 .088553 2.74 0.006 .0337745

APNEA-present .2515853 .1047152 2.40 0.016 .0033304

Cognitive -6.162635 .3976206 -15.50 0.000 -.5571531

Sex 5.863727 .6685324 8.77 0.000 .1187949

Education-level -1.361859 .0772691 -17.62 0.000 -.2834705

Age .7065377 .0693076 10.19 0.000 .1405348

------------------------------------------------------------------------------------

Test_B

DIABETES 6.223344 2.017443 3.08 0.002 .0489179

BDI-total .424836 .1260282 3.37 0.001 .0551442

BMI .7884172 .2869344 2.75 0.006 .0441943

APNEA-present .818319 .3393592 2.41 0.016 .0043578

Cognitive -20.04489 1.073385 -18.67 0.000 -.7290418

Sex 19.07265 2.060045 9.26 0.000 .1554446

Education-level -4.429652 .2144735 -20.65 0.000 -.3709247

Age 2.29812 .209628 10.96 0.000 .1838916

------------------------------------------------------------------------------------

Digits-Forward

DIABETES -.0909007 .0296891 -3.06 0.002 -.0324707

BDI-total -.0062053 .0018518 -3.35 0.001 -.0366036

BMI -.0115159 .0042077 -2.74 0.006 -.0293353

APNEA-present -.0119527 .0049796 -2.40 0.016 -.0028927

Cognitive .292784 .0186298 15.72 0.000 .4839235

Sex -.278583 .0325507 -8.56 0.000 -.1031811

Education-level .0647013 .0041287 15.67 0.000 .2462124

Age -.0335673 .0033032 -10.16 0.000 -.1220636

------------------------------------------------------------------------------------

Digits-Backward

DIABETES -.0973623 .0316829 -3.07 0.002 -.0382795

BDI-total -.0066464 .0019743 -3.37 0.001 -.0431517

BMI -.0123345 .004477 -2.76 0.006 -.0345832

APNEA-present -.0128023 .0053216 -2.41 0.016 -.0034101

Cognitive .3135962 .0181067 17.32 0.000 .5704934

Sex -.2983858 .0338224 -8.82 0.000 -.1216393

Education-level .0693005 .0039647 17.48 0.000 .2902578

Age -.0359534 .0034189 -10.52 0.000 -.1438998

**Supplementary Info S3**. Complete results for the SEM obtained in the T2DM subsample

**Direct effects Coef. Std. Err. z P> z Std. Coef.**

--------------------------------------------------------------------------------------

***Structural***

BDI-total

DURA-TM2DM .0514897 .4236818 0.12 0.903 .0043532

BMI .2504121 .0881952 2.84 0.005 .103788

Sex 3.636717 .618537 5.88 0.000 .2206339

Education-level -.1702371 .0570772 -2.98 0.003 -.1090405

Age -.1121764 .0624219 -1.80 0.072 -.0665088

------------------------------------------------------------------------------------

BMI

Sex 1.181888 .2574309 4.59 0.000 .1730004

Education-level -.0757961 .0239693 -3.16 0.002 -.1171355

Age -.0680516 .0261553 -2.60 0.009 -.0973473

------------------------------------------------------------------------------------

APNEA-present

BMI .0162572 .0040459 4.02 0.000 .1481993

------------------------------------------------------------------------------------

Cognitive

DURA-TM2DM -.2843269 .1155775 -2.46 0.014 -.0867126

BDI-total -.0137355 .0101093 -1.36 0.174 -.0495476

BMI .0063807 .0240461 0.27 0.791 .0095399

Sex -1.264188 .1858116 -6.80 0.000 -.2766647

Education-level .208385 .0215576 9.67 0.000 .4814814

Age -.1227659 .0190001 -6.46 0.000 -.2625636

--------------------------------------------------------------------------------------

Structural

DURA-TM2DM

BMI -.0003088 .0078538 -0.04 0.969 -.0015139

APNEA-present -.0176536 .0719417 -0.25 0.806 -.0094938

Sex -.0813847 .0555264 -1.47 0.143 -.0584011

Age .0138765 .0054427 2.55 0.011 .0973132

--------------------------------------------------------------------------------------

***Measurement***

Letter-P

Cognitive 1 (constrained) .5417137

------------------------------------------------------------------------------------

Animals

Cognitive 1.220697 .0989777 12.33 0.000 .5964362

------------------------------------------------------------------------------------

Test_A

Cognitive -6.521527 .6413958 -10.17 0.000 -.6015391

------------------------------------------------------------------------------------

Test_B

Cognitive -21.18477 1.768005 -11.98 0.000 -.7427476

------------------------------------------------------------------------------------

Digits-Forward

Cognitive .2790035 .0287261 9.71 0.000 .4731223

------------------------------------------------------------------------------------

Digits-Backward

Cognitive .2986148 .0268384 11.13 0.000 .5730718

**Indirect effects Coef. Std. Err. z P> z Std. Coef.**

--------------------------------------------------------------------------------------

***Structural***

BDI-total

BMI -.0000307 .0004707 -0.07 0.948 -.0000127

APNEA-present -.000909 .0083465 -0.11 0.913 -.0000413

Sex .2917323 .127333 2.29 0.022 .0176989

Education-level -.0189779 .0089836 -2.11 0.035 -.0121558

Age -.0163244 .0106547 -1.53 0.125 -.0096786

------------------------------------------------------------------------------------

APNEA-present

Sex .0192142 .0063546 3.02 0.002 .0256385

Education-level -.0012322 .0004959 -2.48 0.013 -.0173594

Age -.0011063 .0005066 -2.18 0.029 -.0144268

------------------------------------------------------------------------------------

Cognitive

DURA-TM2DM -.0007072 .0058427 -0.12 0.904 -.0002157

BMI -.0032697 .0035657 -0.92 0.359 -.0048885

APNEA-present .0050319 .0206074 0.24 0.807 .0008253

Sex -.0230777 .0506781 -0.46 0.649 -.0050505

Education-level .0021025 .0026418 0.80 0.426 .0048579

Age -.0026262 .0030976 -0.85 0.397 -.0056167

--------------------------------------------------------------------------------------

DURA-TM2DM

BMI -.000287 .0011717 -0.24 0.807 -.001407

Sex -.0007042 .0091217 -0.08 0.938 -.0005053

Education-level .0000452 .0005851 0.08 0.938 .0003421

Age .0000405 .0005254 0.08 0.938 .0002843

--------------------------------------------------------------------------------------

***Measurement***

Letter-P

DURA-TM2DM -.2850341 .1157292 -2.46 0.014 -.0470902

BDI-total -.0137355 .0101093 -1.36 0.174 -.0268406

BMI .003111 .0240771 0.13 0.897 .0025197

APNEA-present .0050319 .0206074 0.24 0.807 .0004471

Sex -1.287266 .1814988 -7.09 0.000 -.152609

Education-level .2104875 .0216127 9.74 0.000 .2634566

Age -.1253921 .0189634 -6.61 0.000 -.145277

------------------------------------------------------------------------------------

Animals

DURA-TM2DM -.3479402 .1405944 -2.47 0.013 -.0518472

BDI-total -.0167668 .0123083 -1.36 0.173 -.029552

BMI .0037976 .0294194 0.13 0.897 .0027742

APNEA-present .0061424 .0251542 0.24 0.807 .0004922

Sex -1.571361 .218176 -7.20 0.000 -.1680251

Education-level .2569414 .0232505 11.05 0.000 .2900703

Age -.1530657 .0222014 -6.89 0.000 -.1599525

------------------------------------------------------------------------------------

Test_A

DURA-TM2DM 1.858858 .7474026 2.49 0.013 .0522907

BDI-total .0895762 .0659983 1.36 0.175 .0298048

BMI -.0202887 .1571205 -0.13 0.897 -.0027979

APNEA-present -.0328156 .1343788 -0.24 0.807 -.0004964

Sex 8.394938 1.186654 7.07 0.000 .1694627

Education-level -1.3727 .1223351 -11.22 0.000 -.2925521

Age .817748 .1185969 6.90 0.000 .161321

------------------------------------------------------------------------------------

Test_B

DURA-TM2DM 6.038381 2.421609 2.49 0.013 .0645658

BDI-total .2909825 .2140635 1.36 0.174 .0368013

BMI -.0659064 .5101559 -0.13 0.897 -.0034547

APNEA-present -.1065994 .4365101 -0.24 0.807 -.000613

Sex 27.27043 3.607641 7.56 0.000 .2092433

Education-level -4.459129 .3578171 -12.46 0.000 -.3612273

Age 2.656402 .3614167 7.35 0.000 .1991903

------------------------------------------------------------------------------------

Digits-Forward

DURA-TM2DM -.0795255 .0325024 -2.45 0.014 -.0411277

BDI-total -.0038322 .0028083 -1.36 0.172 -.0234421

BMI .000868 .0067224 0.13 0.897 .0022006

APNEA-present .0014039 .0057499 0.24 0.807 .0003905

Sex -.3591516 .0546533 -6.57 0.000 -.1332857

Education-level .0587267 .0062115 9.45 0.000 .2300979

Age -.0349848 .0053783 -6.50 0.000 -.1268821

------------------------------------------------------------------------------------

Digits-Backward

DURA-TM2DM -.0851154 .0344858 -2.47 0.014 -.0498161

BDI-total -.0041016 .003001 -1.37 0.172 -.0283943

BMI .000929 .007195 0.13 0.897 .0026655

APNEA-present .0015026 .0061535 0.24 0.807 .0004729

Sex -.3843966 .054665 -7.03 0.000 -.161443

Education-level .0628547 .0058382 10.77 0.000 .2787073

Age -.0374439 .0054783 -6.83 0.000 -.1536866

***Total effects*** *Coef. Std. Err. z P> z Std. Coef.*

--------------------------------------------------------------------------------------

***Structural***

BDI-total

DURA-TM2DM .0514897 .4236818 0.12 0.903 .0043532

BMI .2503814 .0881956 2.84 0.005 .1037753

APNEA-present -.000909 .0083465 -0.11 0.913 -.0000413

Sex 3.928449 .6122009 6.42 0.000 .2383329

Education-level -.1892151 .0570012 -3.32 0.001 -.1211963

Age -.1285007 .0622001 -2.07 0.039 -.0761874

------------------------------------------------------------------------------------

BMI

Sex 1.181888 .2574309 4.59 0.000 .1730004

Education-level -.0757961 .0239693 -3.16 0.002 -.1171355

Age -.0680516 .0261553 -2.60 0.009 -.0973473

------------------------------------------------------------------------------------

APNEA-present

BMI .0162572 .0040459 4.02 0.000 .1481993

Sex .0192142 .0063546 3.02 0.002 .0256385

Education-level -.0012322 .0004959 -2.48 0.013 -.0173594

Age -.0011063 .0005066 -2.18 0.029 -.0144268

------------------------------------------------------------------------------------

Cognitive

DURA-TM2DM -.2850341 .1157292 -2.46 0.014 -.0869283

BDI-total -.0137355 .0101093 -1.36 0.174 -.0495476

BMI .003111 .0240771 0.13 0.897 .0046513

APNEA-present .0050319 .0206074 0.24 0.807 .0008253

Sex -1.287266 .1814988 -7.09 0.000 -.2817152

Education-level .2104875 .0216127 9.74 0.000 .4863393

Age -.1253921 .0189634 -6.61 0.000 -.2681803

--------------------------------------------------------------------------------------

DURA-TM2DM

BMI -.0005958 .0077168 -0.08 0.938 -.0029208

APNEA-present -.0176536 .0719417 -0.25 0.806 -.0094938

Sex -.0820889 .0545261 -1.51 0.132 -.0589064

Education-level .0000452 .0005851 0.08 0.938 .0003421

Age .013917 .0054231 2.57 0.010 .0975975

--------------------------------------------------------------------------------------

***Measurement***

Letter-P

DURA-TM2DM -.2850341 .1157292 -2.46 0.014 -.0470902

BDI-total -.0137355 .0101093 -1.36 0.174 -.0268406

BMI .003111 .0240771 0.13 0.897 .0025197

APNEA-present .0050319 .0206074 0.24 0.807 .0004471

Cognitive 1 (constrained) .5417137

Sex -1.287266 .1814988 -7.09 0.000 -.152609

Education-level .2104875 .0216127 9.74 0.000 .2634566

Age -.1253921 .0189634 -6.61 0.000 -.145277

------------------------------------------------------------------------------------

Animals

DURA-TM2DM -.3479402 .1405944 -2.47 0.013 -.0518472

BDI-total -.0167668 .0123083 -1.36 0.173 -.029552

BMI .0037976 .0294194 0.13 0.897 .0027742

APNEA-present .0061424 .0251542 0.24 0.807 .0004922

Cognitive 1.220697 .0989777 12.33 0.000 .5964362

Sex -1.571361 .218176 -7.20 0.000 -.1680251

Education-level .2569414 .0232505 11.05 0.000 .2900703

Age -.1530657 .0222014 -6.89 0.000 -.1599525

------------------------------------------------------------------------------------

Test_A

DURA-TM2DM 1.858858 .7474026 2.49 0.013 .0522907

BDI-total .0895762 .0659983 1.36 0.175 .0298048

BMI -.0202887 .1571205 -0.13 0.897 -.0027979

APNEA-present -.0328156 .1343788 -0.24 0.807 -.0004964

Cognitive -6.521527 .6413958 -10.17 0.000 -.6015391

Sex 8.394938 1.186654 7.07 0.000 .1694627

Education-level -1.3727 .1223351 -11.22 0.000 -.2925521

Age .817748 .1185969 6.90 0.000 .161321

------------------------------------------------------------------------------------

Test_B

DURA-TM2DM 6.038381 2.421609 2.49 0.013 .0645658

BDI-total .2909825 .2140635 1.36 0.174 .0368013

BMI -.0659064 .5101559 -0.13 0.897 -.0034547

APNEA-present -.1065994 .4365101 -0.24 0.807 -.000613

Cognitive -21.18477 1.768005 -11.98 0.000 -.7427476

Sex 27.27043 3.607641 7.56 0.000 .2092433

Education-level -4.459129 .3578171 -12.46 0.000 -.3612273

Age 2.656402 .3614167 7.35 0.000 .1991903

------------------------------------------------------------------------------------

Digits-Forward

DURA-TM2DM -.0795255 .0325024 -2.45 0.014 -.0411277

BDI-total -.0038322 .0028083 -1.36 0.172 -.0234421

BMI .000868 .0067224 0.13 0.897 .0022006

APNEA-present .0014039 .0057499 0.24 0.807 .0003905

Cognitive .2790035 .0287261 9.71 0.000 .4731223

Sex -.3591516 .0546533 -6.57 0.000 -.1332857

Education-level .0587267 .0062115 9.45 0.000 .2300979

Age -.0349848 .0053783 -6.50 0.000 -.1268821

------------------------------------------------------------------------------------

Digits-Backward

DURA-TM2DM -.0851154 .0344858 -2.47 0.014 -.0498161

BDI-total -.0041016 .003001 -1.37 0.172 -.0283943

BMI .000929 .007195 0.13 0.897 .0026655

APNEA-present .0015026 .0061535 0.24 0.807 .0004729

Cognitive .2986148 .0268384 11.13 0.000 .5730718

Sex -.3843966 .054665 -7.03 0.000 -.161443

Education-level .0628547 .0058382 10.77 0.000 .2787073

Age -.0374439 .0054783 -6.83 0.000 -.1536866
